# Timely and Blood-Based Multiplex Molecular Profiling of Acute Stroke

**DOI:** 10.3390/life11080816

**Published:** 2021-08-11

**Authors:** Alexandre Dias, Isabel Silva, Inês Mendes Pinto, Luís F. Maia

**Affiliations:** 1 Department of Neurology, Centro Hospitalar Universitário do Porto (CHUPorto), 4099-001 Porto, Portugal; alexandred@ipatimup.pt (A.D.); isabel.silva@ibmc.up.pt (I.S.); 2 Portugal and Ipatimup—Institute of Molecular Pathology and Immunology, University of Porto, 4200-135 Porto, Portugal; 3 Portugal and Molecular Neurobiology, IBMC—Instituto de Biologia Molecular e Celular, University of Porto, 4200-135 Porto, Portugal; 4 International Iberian Nanotechnology Laboratory, 4715-330 Braga, Portugal; 5 Instituto de Ciências Biomédicas Abel Salazar, University of Porto (ICBAS-UP), 4050-313 Porto, Portugal

**Keywords:** stroke, biomarkers, blood, POC devices, biosensors

## Abstract

Stroke is a leading cause of death and disability in the world. To address such a problem, early diagnosis and tailored acute treatment represent one of the major priorities in acute stroke care. Since the efficacy of reperfusion treatments is highly time-dependent, there is a critical need to optimize procedures for faster and more precise diagnosis. We provide a concise review of the most relevant and well-documented blood–protein biomarkers that exhibit greater potential for translational to clinical practice in stroke differential diagnosis and to differentiate ischemic stroke from hemorrhagic stroke, followed by an overview of the most recent point-of-care technological approaches to address this problem. The integration of fluid-based biomarker profiling, using point-of-care biosensors with demographic, clinical, and neuroimaging parameters in multi-dimensional clinical decision-making algorithms, will be the next step in personalized stroke care.

## 1. Introduction

Stroke is a leading cause of death and disability in the world. Stroke incidence ranges between 172.0 and 198.8 per 100,000/year and varies according to sex, with 52% of all strokes occurring in males (male incidence of 193 per 100,000/year and female incidence rate of 177 per 100,000/year) [1,2,3,4]. In 2016 alone, more than 12 million people had a stroke, and 10% of all deaths worldwide were attributed to stroke (>5,500,000).

**Figure 1:**
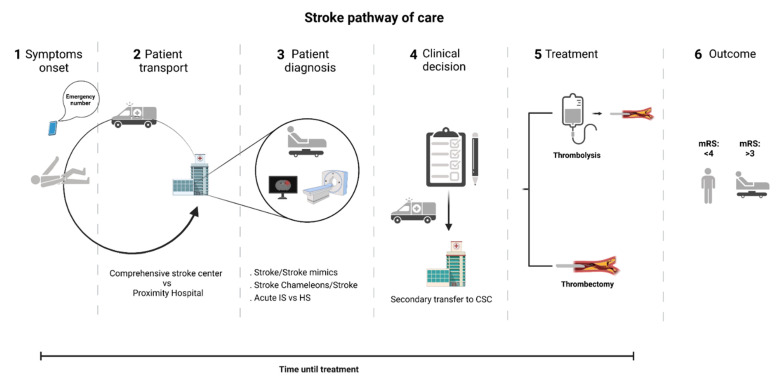
Stroke pathway of care. Currently, the stroke diagnostic strongly relies on skilled clinical and imaging assessment in-hospital, obligating the transport of patients to nearby hospital centers for an initial evaluation. Whenever a patient is considered eligible for thrombectomy, transfer to a comprehensive stroke center may be required. CSC—Comprehensive stroke center; IS—ischemic stroke; HS—hemorrhagic stroke; Mrs—modified Rankin scale.

To tackle such a problem, early diagnosis and tailored acute treatment represent a major priority in acute stroke care [5]. Since the efficacy of reperfusion treatments, such as intravenous thrombolysis (IVT) and, more recently, mechanical thrombectomy (MT), are time-dependent, there is a critical need to optimize procedures for faster and more precise diagnosis [6,7,8,9,10] (Figure 1).

Presently, acute ischemic stroke (IS) diagnosis relies on clinical assessment and neuro and arterial imaging, such as computer tomography (CT) or multimodal brain imaging, but these approaches hold limitations [11,12,13,14]. In fact, brain CT images appear normal in two-thirds of acute IS patients within the first three hours after symptom onset. The magnetic resonance imaging (MRI) approach is more sensitive than CT in acute IS diagnosis, still, 20% of cases may not be noticed [15]. Moreover, MRI is not available in every medical center, and it may not be recommended for unstable or agitated patients or those with a pacemaker [15,16]. Additionally, in the acute context, stroke misdiagnosis can be frequent [17], with a rate of misdiagnosis or failure to recognize a stroke that can reach 26%, even in university centers [18]. These figures are far from reassuring, because, in less skilled settings, the numbers would probably be dramatically higher. Such misdiagnoses occur because: (1) IS can be clinically mistaken for other medical problems (i.e., stroke chameleons) such as dizziness and vertigo, syncope, hypertensive emergency, metabolic or emotional disturbances [19,20], and (2) other medical conditions may be mistaken for acute stroke (i.e., stroke mimics) such as postictal state, systemic infection, syncope, brain tumor, and migraine or toxic–metabolic disturbances [15,21,22]. Such chameleons are frequent causes for missing or delaying acute IS diagnosis that result in lost opportunities for treatment, potentially worsening disability and causing patients death. In addition, stroke mimics, which can represent up to 42% of the acute stroke activations [19,23,24], can lead to inappropriate treatments exposing patients to avoidable side effects.

Together with the confirmation of stroke, identifying the stroke type would also represent an upgrade in acute stroke care. Distinguishing ischemic from hemorrhagic stroke (HS) earlier would allow anticipation of intravenous (iv) thrombolysis, potentially leading to greater chances of treatment success. However, as a CT scan is the gold-standard method for HS diagnosis, portable CT scans would be required to accurately rule out HS in the prehospital setting. For such a purpose, mobile stroke units (MSUs) equipped with CT scanners have been explored, but given the fact that these are scarce and expensive, their widespread clinical use outside the research context is limited [25].

A strategy to improve stroke differential diagnosis (stroke vs. non-stroke) and type of stroke (IS vs. HS) can be achieved by including additional biomarkers to the current practice that include clinical and neuroimaging characteristics. In fact, recent studies have highlighted the potential of blood-derived biomarkers for timely patient triage, therapeutics, and in revealing stroke mechanisms [26,27]. Easily accessible fluid biomarkers can provide an objective evaluation of the real-time panorama, supporting stroke diagnosis or predicting the patient’s outcome, ultimately guiding clinical decisions [11,13,28,29]. Plasma molecular biomarkers, including proteins, metabolites, lipids, and nucleic acids, can be used alone or in combination (panels, scores, or indices), are the potential ideal candidates [30] to detect acute stroke, differentiate IS from HS and stroke mimics, extrapolate the infarct volume, identify the stroke cause, and predict the short/long-term outcome [31]. This would allow a substantial time gain in prehospital settings and, eventually, avoid futile transfers to comprehensive stroke centers (CSCs) [32,33]. Over the past years, several studies identified more than 150 putative molecular biomarkers in patients’ serum for early diagnosis and prognosis [16,33,34,35,36]. Despite several of these having shown great potential, there is currently no blood biomarker for clinical stroke diagnosis.

The demand for high specificity and sensitivity in a heterogeneous disorder with a fast turnaround time at a reasonable cost challenges the recurrent use of biomarkers in clinical settings [37,38,39,40]. Having this in mind, we provide a concise summary of the most relevant and well-documented blood-based protein-biomarkers that exhibit the greater potential for translational to clinical practice in stroke differential diagnosis and to depict stroke type followed by the most recent point-of-care technological approaches to address this problem.

## 2. Circulating Protein Biomarkers for Ischemic Stroke Differential Diagnosis

In this section, we focus on biomarkers that appear to detain potential for distinguishing between IS and other conditions (i.e., stroke mimics). To minimize misdiagnosis and confirm acute IS cases, several plasma-circulating proteins originating from different tissues (mainly from brain cells, blood, and endothelial and mesenchymal cells) have already been described as having the potential to differentiate IS from healthy controls or stroke mimics. Specifically, these include those and originating in brain cells: NR2 peptide [41], S100 calcium binding protein B (S100B) [42], glycogen phosphorylase isoenzyme BB (GPBB) [43], B-type natriuretic peptide (BNP), autoantibodies anti-N-methyl-D-aspartate (NMDA) receptors [44]; those derived from endothelial or mesenchymal cells: matrix metalloproteinase-9 (MMP-9) [34], Parkinson disease protein 7 (PARK7), nucleoside diphosphate kinase A (NDKA) [45]; those found in blood: apolipoprotein A1 unique peptide (APOA1-UP) [46] (Table 1). However, due to the fact of insufficient biomarker performance and/or study limitations, no single protein biomarker has been included in routine clinical practice for acute IS diagnosis [30,47]. To overcome the reduced sensitivity and specificity displayed by some individual markers, researchers have explored multi-marker panel approaches, aimed at a deeper coverage of discrete biological targets from different tissue origins to enhance the diagnostic performance [17]. Despite several panels tested, few exhibited potential for being used in a clinical setting [48]. One of the first studies was conducted Reynolds et al. in which more than 50 plasma proteins were screened in stroke patients, and it was concluded that the top differentially expressed were S100B, B-type neurotrophic growth factor (BNGF), von Willebrand factor (vWF), monocyte chemotactic protein-1 (MCP-1), and MMP-9 [36]. According to the authors, this panel showed a 91.7% sensitivity and a 93% specificity in detecting IS within the first 6 h after symptom onset (Table 2). More recently, a panel of five biomarkers, Eotaxin, epidermal growth factor receptor (EGFR), S100A12, metalloproteinase inhibitor-4 (TIMP-4), and prolactin, was suggested to assist in IS diagnosis within the first 4.5 h after symptom onset, with a 90% sensitivity and an 84% specificity (Table 2) [22]. Additionally, in the BRAIN study, which recruited 1146 patients to test a panel of four biomarkers (i.e., S100B, MMP-9, D-dimer, and BNP), the authors found a panel with 91% sensitivity and 45% specificity for acute IS detection in the first 3 h after symptom onset. The biomarker data were included in a logistic regression model that improved the diagnostic accuracy when compared to early non-contrast CT alone, suggesting that the panel added significant information for acute IS patient’s management [49]. Although available evidence is not yet robust enough to allow for the use of blood biomarkers in current clinical practice, several lines of evidence support that the combination of biomarkers into a panel improves sensitivity and specificity in acute IS diagnosis [17,50]. Considering the rough prediction of brain neuron loss in IS patients (1.9 million each minute) [51], the inclusion of biomarker panels in the diagnostic process that can accelerate and improve stroke diagnosis may reduce the time to treatment, decrease neuronal loss and, ultimately, improve patient outcome [52].

## 3. Circulating Protein Biomarkers to Differentiate Acute IS from HS

An early distinction of IS from HS in the pre-hospital setting could lead to a major improvement in acute stroke care, as it would allow the anticipation of iv thrombolysis and direct acute stroke patients to the most appropriate stroke center. For such a purpose, blood-based biomarkers are the most promising candidates. Though there is no single blood biomarker in use in clinical practice, there are several candidates with the potential to differentiate acute IS from HS. Plasma or serum glial fibrillary acidic protein (GFAP) has been the most consistent candidate [53,54,55,56,57]. This cytoskeletal protein, predominantly expressed in astrocytes, is not released into the bloodstream under physiological conditions and increases earlier and more prominently in HS compared to IS or stroke mimics [26,58,59]. The most relevant blood biomarkers that may distinguish acute IS from HS and that originate in brain cells are GFAP [56], S100B [60], and ubiquitin carboxy-terminal hydrolase-L1 (UCH-L1) [61]; that derive from endothelial cells or mesenchymal cells, receptor for advanced glycation end product (sRAGE) [62,63]; found in the blood, such as the plasmatic retinol-binding protein 4 (RBP4) [27,55] (Table 3). Although some works suggest these candidates are suitable biomarkers for being used in the clinical setting, more research is required to attain that objective. Several other individual biomarkers were tested but were weak candidates, lacking discriminative capacity [27,64,65,66]. Taking into account the complexity of the brain tissue and the numerous proteins that are secreted into the bloodstream during a stroke [16,67], individual biomarkers do not exhibit enough sensitivity/specificity for use in a clinical context. To overcome these obstacles, several research groups analyzed a biomarker panel approach [22,27,54,55,62,65,66,68]. In fact, the combination of proteins that individually did not distinguish acute IS from HS has resulted in a greater capacity to distinguish IS from HS. In Table 4 we depicted the biomarker panels that have been assessed and achieved some accuracy to differentiate between acute IS from HS. In a recent study with 189 stroke patients, Bustamante et al. tested a panel, including NT-proBNP and RBP4, that identified hemorrhagic stroke cases with 29.7% sensitivity and 100% specificity. However, when the diagnostic was performed following a two-step approach, in which the patients with high levels of GFAP (>0.3 ng/mL) were removed first, and the NT-proBNP and RBP-4 panel was used to discriminate the remaining, sensitivity increased to 51.5% with 100% specificity [27]. In another study, a biomarker panel composed of sRAGE and S100B was able to distinguish acute IS vs. HS and to improve stroke diagnosis when compared with the biomarkers alone. In this case, the panel containing both biomarkers worked as a rapid blood test, especially efficiently within the first hours after stroke symptom onset [62] (Table 4). Many other panels have been explored, but with insufficient precision in acute IS/HS differentiation [15,37,38,68,69].

## 4. Conventional and Point-of-Care Technologies

The successful management of stroke is improved with its timely recognition even in pre-hospital settings (e.g., ambulance) [70]. Yet, currently used clinical methodologies, such as enzyme-linked immunosorbent assay (ELISA), mass spectrometry, electrochemiluminescence, immunoturbidimetry, and nephelometry, require sample pre-processing, complex laboratory instrumentation, and long testing procedures that overall limit the possibility to provide fast molecular profiles and disease management in decentralized settings [71,72]. Therefore, the need for novel technologies to deliver personalized information on-site for integration into a time-sensitive workflow has become critical.

**Figure 2:**
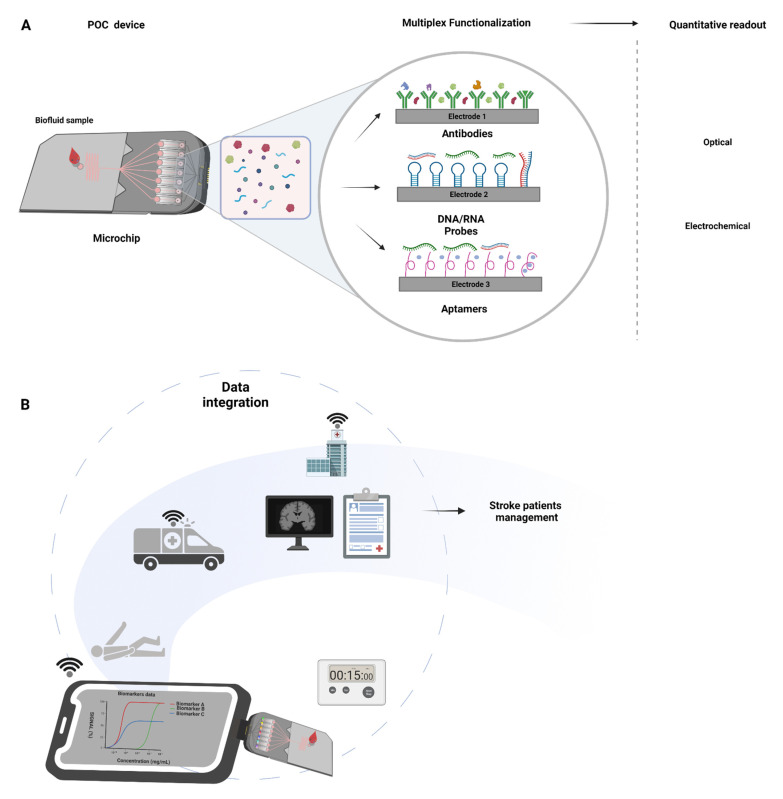
Point-of-care microchips integration in stroke management: (**A**) Figure depicting the general mechanism of microchips. These have several electrodes that confer multiplexing capability and can be functionalized with different antibodies, DNA/RNA probes, and/or aptamers. Examples of methodologies for fast signal acquisition (<15 min) include optical and electrochemical systems. (**B**) Stroke POC device application in stroke pathway of care. Portable, direct blood measurement, multiple markers, signal acquisition in <15 min. Portability and ease to use allows for implementation in ambulatory units at the scene or in-hospital. Wi-Fi data transfer to secure electronic health records allows for biomarker data integration with the clinical and neuroimaging data for an individualized diagnosis and personalized care.

In this context, portable and easy to use point-of-care (POC) biosensor technologies are desirable to enable time-sensitive diagnostics and treatments, preferably during the patient’s first encounter with the paramedics or physicians. By definition, POC analytical platforms are portable, affordable, selective, sensitive, quantitative (or at least semi-quantitative), and easy-to-use, allowing its direct incorporation in mobile units (e.g., emergency ambulances) or in-hospital settings, without the requirements of biological sample processing, central-laboratory long testing, and complex data analysis procedures [73,74]. The biosensor functionality and applicability relies on two major components: an immobilized recognition element designed to target a specific biomarker (e.g., antibodies, aptamers, enzymes) and a transducer that converts the molecular interaction events (e.g., biomarker–antibody) into a measurable signal (e.g., electrochemical, optical) allowing the extrapolation of the biomarker concentration in the study sample (Figure 2A) [75]. There are several commercially available biosensing POC technologies currently used in hospital care for fast (<10–15 min) stroke-related biomarker measurement in the blood (see Table 5). Some examples include bedside and FDA-approved B-type natriuretic peptide (BNP) immunosensor POC platforms (e.g., Abbott AxSYM^®^ BNP, Alere Triage^®^ BNP, and i-STAT BNP) for use on suspected ischemic stroke patients [74,76]; TBI Check^®^ for combinatorial analysis of biomarkers H-FABP, and GFAP for use in brain injury or mild traumatic brain injury (mTBI) detection; the POCs based on fluid phase enzymatic activities or semi-solid phase bioluminescence for plasma NSE monitoring [77]; the lateral flow POC technology detection of c-Fn [78,79]. However, given the heterogeneity of the pathophysiological processes in stroke, a single biomarker has been shown not to be sufficient to reflect the underlying complexity [17]. This has kindled interest in the use of multiple molecular features in the form of proteins, RNA, metabolites, lipids, and others for improved stroke diagnostics sensitivity and specificity [17]. In this context, new miniaturized biosensing POC prototypes are under development for fast multiplex monitoring of different biochemical and genetic pathways potentially associated with stroke (e.g., MMP9, TNF-α, IL6, S100B, GFAP, microRNA) in minimally invasive blood samples [80,81,82,83,84] (see Table 6). Despite recent technological advances, only a few innovative analytical platforms are capable of multi-biomarker panel profiling towards the potential development of tailored POCT stroke devices. These include programmable electrochemical and optical interfaces for protein and genetic biomarkers sensing, such as an electrochemical impedance spectroscopy (EIS) system, the ELFI (electro-lateral-flow-immunoassay), and the Stack pad analytical platforms, which enable quantitative and multiplex detection (see Figure 2A and Table 6). The EIS system comprises a sensing component with multiple functionalized electrodes to transduce biomarkers concentrations in considerably small blood sample volumes into electrochemical signals. The second combines a flow test strip with a screen-printed electrode and relies on immune-electroactive nanobeads for ultrasensitive recognition of biomarkers, and the results are measured electrochemically [85,86,87]. The third platform is based on stacked and differently functionalized membranes, from which a measurable signal is obtained via biomarker migration linked, for example, to an antibody conjugated with enzyme horseradish peroxidase [88,89,90]. The short time of analysis provided by any of these technological configurations allows the molecular characterization of the patient prior to hospital admission and a subsequent clinical decision within the time window for treatment (Figure 2B). In recent years, the technological developments in the field of biosensors and wireless communications have enabled the testing of low sample volumes without significant technical requirements. The key to multiplex testing lies in microchip technology capable of running multiple biomarker component-resolved diagnostic assays rapidly and cost effectively. In this context, electrochemical and optical chips play a key role due to the fact of their ability to be adapted to small hand-held devices at a low cost and capacity of integration with microfluidic platforms for advanced and ultra-sensitive high-throughput analytics [91,92,93]. In these settings, in a single step, the comprehensive biomarker panel profile of a patient can be potentially measured using a single blood drop, allowing integration and analysis of multiple biomarkers as well as other meta-data from the patient (i.e., electronic health record) and timely clinical decision making (Figure 2B).

Overall, these analytical platforms represent a promising set-up to allow for quantitative, multiplex biomarker detection (e.g., proteins, nucleic acids, metabolites, lipids); yet, its clinical validation and applicability in stroke diagnostics remains to be seen.

## 5. Future Perspectives

**Figure 3:**
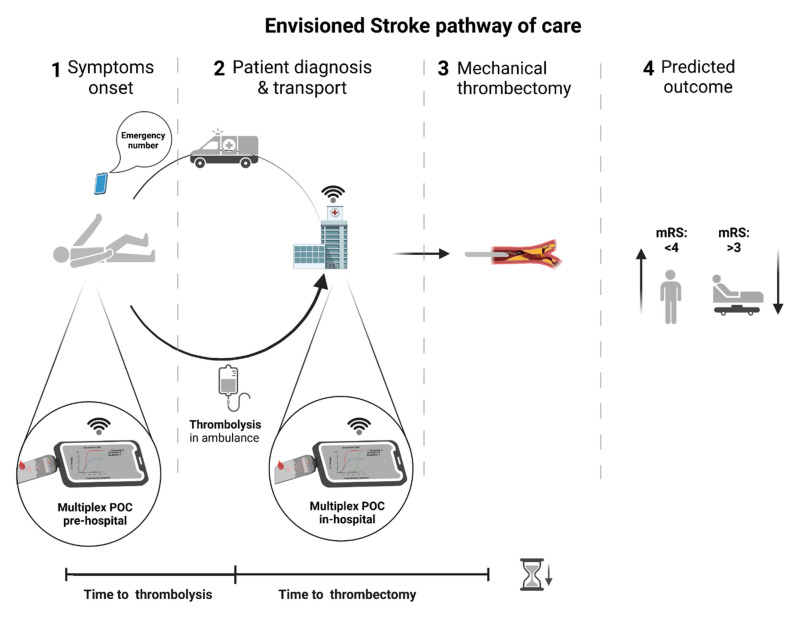
Envisioned stroke pathway of care. The inclusion of fast and specific biomarker assessment using POC devices at the pre-hospital setting would accelerate diagnosis and eventually allow starting thrombolysis in the ambulance. In-hospital, the inclusion of biomarker assessment using POC devices would improve differential diagnosis and identify acute IS cases decreasing misdiagnosis (i.e., chameleons and stroke mimics) leading to accurate and timely patient treatment, better outcomes, and health resources optimization. POC—point-of-care; mRS—modified Rankin scale.

As stroke diagnosis and treatment efficacy strongly rely on time, the incorporation of biomarker-based POC technologies in the stroke pathway of care will significantly improve patient care (Figure 3). Stroke-related biomarker research is a growing field, with new biomarkers featuring different pathophysiological mechanisms being discovered regularly. In order to fully make use of biomarker measurement for the integration of POCT devices in stroke care, there is a need to make use of miniaturized analytical tools capable of fast readout, multiplex capability, and remote data transfer. Presently, the offer of an extensive and systematic application of circulating proteins as biomarkers in acute stroke differential diagnosis is still not available in clinical settings [33]. Still, several candidates exhibited potential in stroke vs. non-stroke and acute IS vs. HS differential diagnosis or even in prognostic evaluation [16,27,34,35,36,38,110].

Assuming that biomarker panels under investigation achieve the diagnostic performance for clinical use and a POC device is validated to complement existing diagnostic and stratification strategies, the traditional pathway of care would be reconfigured (Figure 3) with a clear impact on the following clinical scenarios:

- The recognition of stroke mimics would become more efficient. The frequency of these can vary between 15% and 42% and entail an inappropriate use of the available stroke facilities leading to additional costs and a delayed diagnosis of the actual disease [19,23,24]. Even more, the administration of thrombolytic medication in wrongly diagnosed patients may lead to undesirable side effects such as intracranial hemorrhage [111];
- Stroke chameleons’ recognition at patient admission to the hospital would be more sensitive and specific. The frequency of these can vary between 2% and 26% [18]. The problem of chameleons resides in the lack of proper treatment of stroke patients during the hyper-acute settings due to the fact of missing diagnosis, lowering the chance to administer thrombolytic medication or to undergo mechanical thrombectomy as well as to receive suitable secondary prevention. Consequently, stroke chameleon patients have the worst outcomes at 12 months [10];
- The reperfusion treatments would be hastened. Thrombolytic iv treatment would start right after the first encounter of the paramedic team with the patient, saving over 15 min, depending on the time and distance from the scene to hospital [70], at a significantly lower cost than specialized stroke ambulances with portable imaging devices [112];
- Biomarkers able to anticipate successful recanalization (e.g., reduced levels of inflammation-associated α2-antiplasmin and thrombin-activatable fibrinolysis inhibitor (TAFI) or C-Reactive Protein) [113,114,115], could guide adjuvant therapies (e.g., growth factors administration) [116] to improve the efficacy of thrombolytic iv treatment in centers where mechanical thrombectomy is not readily available or when thrombectomy is not recommended (distal clots with low NIHSS at presentation and high pretreatment modified Rankin scale) [117]. In addition, biomarkers that predict the risk of hemorrhagic transformation after iv thrombolysis or mechanical recanalization (e.g., cellular Fibronectin (c-Fn)) could be measured with POC diagnostic platforms preventing damaging interventions [79];
- The identification of the stroke subtype in the pre-hospital setting would be more sensitive. For instance, the earlier recognition of patients with large vessel occlusions would be possible, and the transport for a comprehensive stroke center would be ensured (Figure 3), reducing the need for secondary transfers (saving up to 100 min) and reducing the time from symptoms onset to mechanical thrombectomy in a timely fashion [32]. The inverse is also applicable to the identification of cases in which mechanical thrombectomy would not be a valuable strategy and would save time and avoid the inappropriate use of comprehensive stroke facilities [118].

The current technological point opens perspectives on the implementation of pilot studies using blood-based biomarkers within the current clinical pathways of care to assess their role in improving diagnostic acuity and differential diagnosis. Moreover, sex-related biomarker differences need to be explored in future studies given the different patterns of protein expression, such as sRAGE, that are less expressed in males and positively associated with stroke.

Finally, the integration of fluid biomarker profiling with the demographic, clinical, and neuroimaging individual parameters in multi-dimensional algorithms capable of clinical decision making will be the next step in the generation of individual patient composite scores for acute IS diagnosis or stratification for personalized treatment [119].

## Figures and Tables

**Table 1:** Biomarkers to differentiate acute ischemic stroke from non-IS conditions.

Biomarker Origin	Protein	Biomarker Level in IS	Biomarker Level in Control	Cut-Off Point	Sensitivity	Specificity	AUC	Study Sample	Reference
Brain Cells	NR2	5.4 (0.1–62.7) ng/mL	0.3 (0.02–1.1) ng/mL	1 ng/mL	92.1%	96.5%	0.92	Combined stroke mimics and healthy controls	[41]
	S100B	N/A	N/A	39.9 pg/mL	76.5%	82.7%	0.87	Non-stroke controls	[42]
	GPBB	46.3 (±38.6) ng/mL	4.1 (±7.6) ng/mL	7.0 ng/mL	93.0%	93.0%	0.96	Non-stroke controls	[43]
	BNP	90.8 (±156.4) pg/mL	11.3 (±6.1) pg/mL	N/A	N/A	N/A	0.69	Healthy and stroke mimics	[34]
	Anti-NMDA (NR2A/2B ab)	5.0 (3.2–7.2) ng/mL	1.5 (1.0–1.9) ng/mL	2.0 ng/mL	97.0%	98.0%	0.99	Healthy controls	[44]
Brain Cells, Endothelium/Matrix, Blood	MMP-9	242.1 (±242.6) ng/mL	211.2 (±184.8) ng/mL	N/A	N/A	N/A	0.55	Healthy and stroke mimics	[34]
	PARK 7	N/A	N/A	14.2 ng/mL	58.0%	90.0%	0.88	Healthy controls	[45]
	NDKA	N/A	N/A	22.5 ng/mL	67.0%	89.9%	0.94	Healthy controls	[45]
Blood	APOA1-UP/LRP	1.3 (IQR 0.4)	2.1 (IQR 0.4)	<1.8	90.6%	97.1%	0.98	Non-stroke controls	[46]

NR2—N-methyl-D-aspartate receptor subunits peptide; S100B—S100 calcium binding protein B; GPBB—glycogen phosphorylase isoenzyme BB; BNP—B-type natriuretic peptide; anti-NMDA—autoantibodies anti-N-methyl-D-aspartate receptors; MMP-9—matrix metalloproteinase-9; PARK 7—Parkinson disease protein 7; NDKA—nucleoside diphosphate kinase A; APOA1-UP/LRP—apolipoprotein A1 unique peptide; N/A—not available; IS biomarker value was higher than controls except indicated by “<”; IQR—interquartile range.

**Table 2:** Biomarker panels to differentiate acute ischemic stroke from non-IS conditions.

Biomarker Origin	Proteins	Biomarker Level in IS	Biomarker Level in Control	Cut-Off Point	Sensitivity	Specificity	AUC	Study Sample	Reference
Brain Cells, Endothelium/Matrix, Blood	MMP9	N/A	N/A	N/A	91.7%	93.0%	0.99	Healthy controls	[36]
	BNGF								
	vWF								
	MCP-1								
	S-100B								
Brain Cells, Endothelium/Matrix	Eotaxin	N/A	N/A	N/A	N/A		0.92	Stroke mimics	[22]
	EGFR								
	S100A12								
	TIMP-4								
	Prolactin								
Brain Cells, Endothelium/Matrix, Blood	BNP	90.8 (±156.4) pg/mL	11.3 (±6.1) pg/mL	N/A	91.0%	21.5%	N/A	Healthy controls and stroke mimics	[34]
	D-dimer	888.1 (±1289) ng/mL	188.6 (±113.8) ng/mL						
	MMP9	242.1 (±242.6) ng/mL	211.2 (±184.8) ng/mL						
	S100B	103.1 (±13.6) pg/mL	188.6 (±147.1) pg/mL						
Brain Cells, Endothelium/Matrix, Blood	IL-6	4.0 (0.8–12.3) pg/mL	1.2 (0.0–2.4) pg/mL	-	N/A	N/A	0.75	Stroke mimics	[48]
	S100B	63.3 (29.7–122.8) ng/mL	33.8 (15.4–60.8) ng/mL						
	MMP-9	30.4 (0–115.2) pg/mL	2.3 (0.0–20.6) pg/mL						

MMP-9—matrix metalloproteinase-9; BNGF—B-type neurotrophic growth factor; vWF—von Willebrand factor; MCP-1—monocyte chemotactic protein-1; S100B—S100 calcium binding protein B; EGFR—epidermal growth factor receptor; S100A12—calcium binding protein A12; TIMP-4—metalloproteinase inhibitor-4; BNP—B-type natriuretic peptide; IL-6—interleukin-6; N/A—not available.

**Table 3:** Biomarkers to differentiate acute ischemic stroke from hemorrhagic stroke.

Biomarker Origin	Protein	Biomarker Level in IS	Biomarker Level in HS	Cut-Off Point	Sensitivity	Specificity	AUC	Reference
Brain Cells	GFAP	0.08 (0.02–0.14) ng/mL	1.91 (0.41–17.7) ng/mL	0.30 ng/mL	84.2%	96.3%	0.91	[56]
	S100B	61.7 (±37.3) pg/mL	161.2 (±79.7) pg/mL	67.0 pg/mL	95.7%	70.4%	0.90	[60]
	UCH-L1	338.0 pg/mL	401.0 pg/mL	291.0 pg/mL	73%	45.0%	0.59	[61]
Endothelium/Matrix	sRAGE	1.0 ng/mL	0.8 ng/mL	<0.97 ng/mL	NA	NA	NA	[62]
Blood	RBP4	59.8 (±12.3) µg/mL	36.9 (±14.7) µg/mL	61.0 µg/mL	68.4%	84.0%	NA	[55]

GFAP—glial fibrillary acidic protein; S100B—S100 calcium binding protein B; UCH-L1—ubiquitin carboxy-terminal hydrolase-L1; sRAGE—receptor for advanced glycation end product; RBP4—retinol-binding protein 4; N/A—not available; HS biomarker value was higher than IS except when indicated by “<”.

**Table 4:** Biomarker panels to differentiate acute ischemic stroke from hemorrhagic stroke.

Biomarkers Origin	Proteins	Biomarker Level in Ischemic Stroke	Biomarker Level in Hemorrhagic Stroke	Cut-Off Point	Sensitivity	Specificity	AUC	Reference
Brain Cells, Endothelium/Matrix, Blood	RBP-4	29.2 (25.1–35.7) μg/mL	34.4 (26.0–40.0) μg/mL	38.0 μg/mL	51.5%	100%	N/A	[27]
	NT-proBNP	0.8 (0.2–2.4) ng/mL	0.4 (0.2–0.7) ng/mL	1.3 ng/mL				
	GFAP	186.3 (132.8–280.2) pg/mL	1699.6 (411.1–10,145.4) pg/mL	325 pg/mL				
	sRAGE	1.0 ng/mL	0.8 ng/mL	<0.9 ng/mL	22.7%	80.2%	0.76	[62]
	S100B	58.7 pg/mL	107.7 pg/mL	96.0 pg/mL				

RBP4—retinol-binding protein 4; NT-proBNP—N-terminal pro b-type natriuretic peptide; GFAP—glial fibrillary acidic protein; sRAGE—receptor for advanced glycation end product; S100B—S100 calcium binding protein B; N/A—not available; HS biomarker value was higher than IS except when indicated by “<”.

**Table 5:** Commercially available and in clinical trials point-of-care technologies (POCTs).

POC Device	Analytical Platform	Blood Biomarkers	Application	Reference
Hemochron^®^ Junior	Optical	ACT-LR, ACT, PT, Citrate PT, APTT, and Citrate APTT	Pre- and In-hospital	[94]
PocH-100i Hematology Analyzer	Hydrodynamics/Impedance	Full blood cell count	Pre- and In-hospital	[95]
i-STAT	Electrochemical	Blood gases, electrolytes, metabolites, and coagulation	Pre- and In-hospital	[96]
Reflotron^®^ plus analyzer	Optical	c-glutamyltransferase, p-amylase, glucose	Pre- and In-hospital	[97]
AxSYM^®^ BNP	Optical	BNP	In/Post-hospital	[98]
Triage^®^ BNP	Optical	BNP	In/Post-hospital	[99]
iSTAT BNP	Electrochemical	BNP	In/Post-hospital	[100]
TBI Check^®^	N/A *	H-FABP and GFAP	Pre- and In-hospital	[101]
Prediction Sciences LLC	Optical	c-Fn	In-hospital	[102,103]
ReSTTM	N/A *	Immune response	In-hospital	[104,105]
SMARTChip	Electrochemical	Purines	In-hospital	[106]

ACT-LR—activated clotting time in low-range heparin plasma concentrations; ACT—activated clotting time; PT—prothrombin; citrate PT—citrate prothrombin; APTT—activated partial thromboplastin time; citrate APTT—citrate activated partial thromboplastin time; BNP—B-type natriuretic peptide; H-FABP—heart-type fatty acid binding protein; GFAP—glial fibrillary acidic protein; NSE—neuron-specific enolase; c-Fn—cellular fibronectin; N/A—not available; * Technical details not disclosed.

**Table 6:** Point-of-care technologies under development.

POC	Modality	Analytical Platform	Multiplex Capacity *	On-Site Analysis	Reference
µPADs	Paper-based system	Optical	≥2 biomarkers	Yes	[73]
Stack Pad	Paper-based system	Optical	>2 biomarkers	Yes	[88,89,90,106]
ELFI	Paper-based system	Electrochemical	>2 biomarkers	Yes	[85,86,87]
EIS-SERS	Paper-based system	Electrochemical and surface-enhanced Raman spectroscopy	≥2 biomarkers	Yes	[107]
EIS	Array-based system	Electrochemical	≥5 biomarkers	Yes	[108,109]
MuitiLab	Microfluidic-based system	Electrochemical	≥8 biomarkers	Yes	[73]
mChip	Microfluidic-based system	Optical	≥5 biomarkers	Yes	[73]

* Reconfiguration capacity for nucleic acids, protein, metabolite and lipids detection.

## Data Availability

Not applicable.
